# Editorial: Exploring adverse drug reactions: monitoring, mechanism, intervention, and resolution

**DOI:** 10.3389/fmed.2026.1798711

**Published:** 2026-02-26

**Authors:** Li Tao, Xin Li, Yunhao Wu, Yunlong Shan

**Affiliations:** 1 Faculty of Medicine, Yangzhou University, Yangzhou, China; 2 Laboratory of Cancer Immunometabolism, Center for Cancer Research, National Cancer Institute, NIH, Frederick, MD, United States; 3 Medical Science and Technology Innovation Center, Shandong First Medical University, Jinan, Shandong, China; 4 State Key Laboratory of Natural Medicines, China Pharmaceutical University, Nanjing, Jiangsu, China

**Keywords:** adverse drug reactions (ADRs), causality attribution, machine learning, precision safety management, proactive pharmacovigilance

Adverse drug reactions (ADRs) continue to challenge health systems worldwide and represent the most important cause of treatment failure and high healthcare cost burden. With the rapid development and adoption of innovative therapies, such as targeted therapies, immunotherapies, biologics, and personalized treatment regimens, the spectrum of ADRs has become increasingly diverse and idiosyncratic, leading to unprecedented levels of clinical uncertainty. Despite advances in active surveillance, there is still a significant unmet need to optimize interventions to prevent or mitigate ADRs. Traditionally, ADRs have been identified and managed appropriately through routine observation and effective symptom control. However, accumulating evidence suggests that increasing ADRs arise from undefinable biological processes, including interindividual variability, delayed onset time patterns, and complex clinical trajectories, *etc*.

This Research Topic gathers different contributions to provide a comprehensive and integrative exploration of ADRs in a wide-ranging clinical setting, spanning mechanistic studies, causality analysis, risk factor identification, monitoring strategies, and emerging predictive approaches, which aims to establish a precision-oriented framework for advancing the proactive management of ADRs. The 15 studies included in this Research Topic collectively demonstrate that modern pharmacovigilance is no longer limited to predefined causal assessment. Instead, it has shifted toward the comprehensive capture and interpretation of complex clinical events, embracing a full continuum of patient-specific responses across various biological contexts.

## Proactive monitoring

This Research Topic highlights the importance of systematically characterizing delayed or atypical ADRs. For example, clinically significant immune-related adverse events with multisystem involvement emerge long after treatment initiation or even following drug discontinuation, underscoring how delayed onset may lead to repeated exposure and life-threatening outcomes if early causality attribution fails. As illustrated by Hao et al., sintilimab-induced colitis progresses to intestinal obstruction and hemorrhage despite initial corticosteroid therapy. Early endoscopic evaluation, timely escalation to biologic agents, and multidisciplinary management are crucial to mitigate severe complications. Vigilant monitoring and individualized treatment plans are essential for improving outcomes in high-risk patients (Hao et al.). Osilodrostat is a novel steroidogenesis inhibitor for the treatment of Cushing's syndrome. However, prolonged endocrine suppression may persist even after the discontinuation of osilodrostat, potentially due to extensive off-target effects (Dzialach et al.). In addition, investigations into postacute COVID-19 vaccination syndrome also highlight the need to establish systematic efforts for long-term and delayed adverse events, challenging the conventional temporal bounds of pharmacovigilance frameworks (Halma and Varon).

Machine learning-based prediction models have a considerable positive impact on proactive risk identification and stratification. Yue et al. recognized gastrointestinal comorbidities, alcohol consumption, and concomitant medication use as key multifactorial, pathology-driven risk factors for semaglutide-associated adverse events. A logistic regression-based nomogram scoring tool was subsequently developed to estimate the risk of semaglutide-induced gastrointestinal adverse events (Yue et al.). Similarly, machine learning algorithms such as XGBoost were employed to stratify key determinants, such as IL-6, distinguishing outcomes between mono- and multimodal treatment strategies in pancreatic cancer patients (Fan et al.). Wang X. et al. evaluated the association between KRT8 expression and the development of immune-related pneumonitis (IRP) via statistical models, including multivariate logistic regression, bootstrap validation, and Bayesian analysis, suggesting that elevated KRT8 expression in tumor tissue may serve as a potential biomarker for predicting IRP in lung adenocarcinoma patients receiving immunotherapy.

## Underlying mechanisms

At the core of biologically relevant heterogeneity lies the molecular interaction between drugs and biological systems that drives diverse adverse events. Several studies in this Research Topic provide a mechanistic dissection of adverse events, unraveling the complex causal chains between drug exposure and clinical manifestations. Zhang Y. et al. revealed that ibrutinib induces auditory cell apoptosis and hearing loss by inhibiting GPR83-AKT signaling. GPR83 overexpression activates AKT to protect auditory cells, while the caspase inhibitor Z-VAD-FMK mitigates ototoxicity. These findings identify GPR83-AKT as a potential target for preventing chemotherapy-induced hearing loss (Zhang Y. et al.).

Rare events such as paroxetine-induced pancreatitis have been confirmed through involuntary rechallenge in the absence of conventional risk factors. Paroxetine may induce pancreatitis through multiple nonexclusive mechanisms. Elevated serotonin levels caused by SERT inhibition can impair β-cell function and disrupt insulin and exocrine secretion. Additionally, reactive catechol metabolites and individual genetic polymorphisms in CYP2D6/CYP2C19 may trigger T-cell-mediated hypersensitivity reactions, further contributing to pancreatic injury (Wu Y. et al.). Tranexamic acid (TXA) is a synthetic lysine analog that inhibits fibrinolysis to reduce perioperative bleeding during posterior lumbar interbody fusion (PLIF). TXA reduces perioperative blood loss in PLIF primarily by inhibiting the fibrinolytic system, stabilizing fibrin clots, protecting coagulation components, and exerting beneficial anti-inflammatory actions without significantly increasing systemic thrombotic risk when used at appropriate doses (Dong et al.).

## Effective interventions

Several studies have demonstrated that natural compounds can simultaneously increase therapeutic efficacy and mitigate toxicity by modulating convergent signaling pathways involved in the immune response. Tao, Chang, et al. demonstrated that cycloastragenol (CAG) inhibits the JAK/STAT5 signaling pathway, reducing the levels of the neutrophil chemotactic cytokines CXCL3 and CCL5, thereby limiting neutrophil infiltration into brain metastases and enhancing the efficacy of radiotherapy. Concurrently, CAG suppresses the IKK/NF-κB pathway, promotes microglia/macrophage polarization toward an anti-inflammatory phenotype, mitigates neuroinflammation, and protects brain function from radiation-induced injury (Tao, Chang, et al.). In addition, homoplantaginin alleviates DSS-induced ulcerative colitis in mice by modulating the MMP9-RLN2 signaling axis, reducing proinflammatory cytokines and immune cell infiltration while protecting the intestinal barrier (Tao, Shao, et al.). Similarly, Folium isatidis and its active ingredients mitigate COPD-related lung lesions by correcting aberrant immune cell infiltration, restoring immune homeostasis, and improving pulmonary function (Wang F. et al.).

## Systematic resolutions

Evidence-based approaches provide the foundation for managing ADRs by identifying risk factors, quantifying incidence, and evaluating intervention efficacy. Wu T. et al. analyzed 1,026 patients receiving methylprednisolone sodium succinate in the treatment for idiopathic sudden sensorineural hearing loss, highlighting evidence-based solutions such as preemptive proton pump inhibitor use for gastrointestinal protection, regular monitoring of blood glucose and blood pressure, individualized dose adjustments and a closed-loop “assessment-intervention-reassessment” management strategy to minimize severe ADRs. Li et al. summarized the best evidence for precision management of EGFR inhibitor-induced skin across cancer types and different EGFR inhibitors, emphasizing early prophylactic measures, patient education, symptom-guided topical or systemic therapy, and the integration of dermatology and oncology teams to prevent progression and maintain therapy adherence. Liu et al. evaluated the safety of gemtuzumab ozogamicin via real-world FDA adverse event reports. The primary risks include hepatic veno-occlusive disease (VOD) and infections secondary to myelosuppression, warranting vigilant pharmacovigilance, organ function monitoring, infection prevention, and individualized dose adjustments to mitigate hematologic and hepatic toxicity. Newly identified signals, such as coagulation disorders and cryptogenic organizing pneumonia (COP), further underscore the need to expand safety monitoring (Liu et al.).

Taken together, these 15 studies illustrate that ADRs should no longer be regarded as unpredictable accidents addressed solely through symptom interventions. Instead, ADRs are biologically traceable and proactively manageable. By leveraging machine learning and artificial intelligence to identify risk factors and biomarkers, mechanistic insights to clarify causality, and evidence-based strategies to translate biological signals into clinical imperatives, modern pharmacovigilance is shifting from passive observation toward proactive management. Importantly, predictive analytics tools and longitudinal monitoring will be essential for achieving the ultimate balance between therapeutic efficacy and safety. In the era of precision medicine, effective safety management must extend across the full therapeutic lifecycle, including postdiscontinuation surveillance, to prevent latent and cumulative health risks.

